# Aliskiren Reduces the Adrenal Zona Glomerulosa Apoptosis and Autophagy in Wistar Rats with 2K1C Hypertension

**DOI:** 10.1155/2020/7684849

**Published:** 2020-10-21

**Authors:** Veronica S. de Matos, Ana L. R. do Nascimento, Priscila G. Pereira, Kíssila Rabelo, Cherley B. V. Andrade, Alan C. N. Moraes, Camila Salata, Victor H. V. de O. Araújo, Bianca Torres Ciambarella, Aline Bonfim Vieira, Luciana Lontro Alves, Jemima F. R. da Silva, Jorge J. de Carvalho

**Affiliations:** ^1^Laboratory of Ultrastructure and Tecidual Biology, Institute of Biology, State University of Rio de Janeiro, Rio de Janeiro, Brazil; ^2^Translational Endocrinology Laboratory, Institute of Biophysics Carlos Chagas Filho, Health Sciences Center, UFRJ, Rio de Janeiro, Brazil; ^3^Institute of Biology, UFF, Rio de Janeiro, RJ, Brazil; ^4^National Commission of Nuclear Energy, CNEN, Rio de Janeiro, Brazil; ^5^Ross University School of Veterinary Medicine, Biomedical Department, Basseterre, Saint Kitts and Nevis

## Abstract

Hypertension is a disease classified as primary or secondary, manifested not only by elevation of blood pressure but also involved in structural and functional changes of target organs. Renal artery stenosis is a leading factor of secondary hypertension, and its progress is associated with overactivation of the renin-angiotensin-aldosterone system (RAAS). Aliskiren is a renin inhibiting drug that suppresses RAAS and culminates in decreased renin release, plasma angiotensin II concentration, and inhibition of aldosterone secretion. In this sense, the aim of the present study was to analyze the structural and ultrastructural morphophysiology of the adrenal glomerular zone, after treatment with aliskiren in Wistar rats with 2K1C hypertension. Parameters as structure and ultrastructure of the adrenal glomerular zone, cellular apoptosis, nuclear cell proliferation, and AT1 receptor expression were analyzed by immunostaining and electron microscopy. Our results showed that the hypertensive animals treated with aliskiren presented a reestablishment of AT1 receptor expression and decrease in apoptosis and autophagy. In addition, treatment with aliskiren improves the cell aspects in the adrenal glomerular zone, evidenced by ultrastructural analysis through preserved nuclei and well-developed mitochondria. Therefore, our evidence suggests that aliskiren has a beneficial effect on the adrenal glomerular zone remodeling in animals with renovascular hypertension.

## 1. Introduction

The adrenal glands synthesize and secrete steroid hormones that are effectors of adaptive responses to oscillations in the organism's internal and external environment, broadly referred as stress. Adrenal steroid hormones serve to modulate a wide range of processes that are central to physiologic responses to stress, including energy metabolism, immune response, electrolyte homeostasis, and fluid balance [1]. To fulfill these tasks, the adrenal cortex is organized into functionally and histologically distinct zones: the outermost zona glomerulosa (ZG), the intermediate zona fasciculata (ZF), and the innermost zona reticularis (ZR), which are responsible for the production of mineralocorticoids, glucocorticoids, and androgens, respectively [2, 3].

The mineralocorticoid aldosterone is a hormone synthesized by cells in the zone of the adrenal cortex. This hormone plays key roles in mammalian physiology, including regulation of electrolyte balance and blood pressure. Aldosterone synthesis and secretion are controlled by the renin-angiotensin-aldosterone system (RAAS) [4]. Extreme release of this hormone causes high blood pressure, inflammation, heart failure, cardiac fibrosis, and kidney injury [5].

High blood pressure is a precondition that leads to the pathological condition called hypertension, which afflicts an estimated 1 billion people and results in 9 million deaths annually. Globally, it is also responsible for 40% of all deaths associated with cardiovascular diseases (CVD) [6]. Arterial hypertension (AH) is a multifactorial clinical condition characterized by sustained rise in blood pressure ≥140/90 mmHg. AH is frequently associated with metabolic disorders, functional alterations, and/or target organ structures, aggravated by the presence of other risk factors [7, 8]. High blood pressure is already a health problem, with a projected prevalence of about one-third of the world's population by 2025 [9].

Renovascular hypertension (RH) is a secondary clinical disease that reaches about 6.8% for renal stenosis in elderly patients. RH is defined as high blood pressure caused by renal hypoperfusion, usually resulting from renal ischemia due to stenotic events triggered by occlusive renal artery damage and activation of the renin-angiotensin-aldosterone (RAAS) system. This hypertensive condition usually accelerates preexisting hypertension and causes chronic kidney disease [10, 11].

Experimental RH was first implemented by Goldblatt [12] in dogs, known as the 2 kidneys 1 clip (2K1C) model. Thus, the 2K1C model has been widely used to experimentally study RH and its local and systemic changes [13–16]. In this model, RH is induced by partial unilateral renal artery occlusion, leading to renin secretion, RAAS over activation, development of arterial hypertension, cardiac hypertrophy, vascular remodeling, and kidney disease [17, 18].

Renal artery stenosis reduces tissue perfusion that results in increased renin release by juxtaglomerular cells in the efferent arteriole. Thus, RAAS over activation culminates in high levels of angiotensin II (Ang II) in serum. Ang II acts on the Ang II type 1 receptor (AT1R) to induce a number of effects that affect the cardiovascular system, including increases in oxidative stress, vasoconstriction, increased blood pressure, and increased adrenal secretion of aldosterone [18–21]. Aldosterone over production causes systemic oxidative stress and increases Ang II binding to AT1R [22, 23]. In this sense, inhibition of RAAS would decrease Ang II levels and, consequently, aldosterone adrenal secretion, reducing local and systemic changes.

Aliskiren is a commercially approved drug, which directs inhibits renin, also leading to a decline in Ang II and aldosterone levels [24]. In addition, unlike angiotensin-converting enzyme (ACE) inhibitors and Ang II receptor blockers, aliskiren neutralizes any compensatory increase in plasma renin by preventing formation of Ang I and Ang II [25]. Aliskiren administration not only has a favorable effect on blood pressure (BP) reduction but also acts on target organs damaged by hypertension, preventing or treating, for example, cardiovascular and renal dysfunction [26, 27].

Based on the above, in this study, we investigated the effects of treatment with aliskiren in the zona glomerulosa of the adrenal gland of rats with renovascular hypertension 2K1C. Therefore, we evaluated tissue and ultrastructure morphology, AT1 receptor levels, apoptotic markers, besides proteins related to autophagy, and mitochondrial biogenesis in the adrenal gland.

## 2. Materials and Methods

### 2.1. Animals and Experimental Groups

All experimental procedures involving animals were approved by the Committee for Ethics in Animal Experimentation of the State University of Rio de Janeiro (CEUA/007/2016). Forty-two-month-old male Wistar rats weighing an average of 180g were placed in cages with free access to standard commercial feed (Nuvilab, Curitiba, Paraná, Brasil) and water. The animals were kept under controlled conditions (temperature 21 ± 2 °C, humidity 60 ± 10%, 12 h inverted light cycle–light/dark, and air replacement cycle 15 min/h).

Animals were divided in four groups (*n* = 10): SHAM, SHAM treated with aliskiren (SHAM + A), 2K1C (hypertensives), and 2K1C treated with aliskiren (2K1C + A). For the experiment, all animals were anesthetized with intraperitoneal injection of ketamine (100 mg/kg) and xylazine (10 mg/kg). During the surgical procedure, the animals of 2K1C and 2K1C + aliskiren groups had their left renal artery partially obstructed with a 0.2 mm silver clip according to the experimental model 2 kidneys 1 clip (2K1C) developed by Goldblatt in 1934 (6). SHAM and SHAM + A groups were submitted to the same surgical procedure, with manipulation of the left renal artery, but the clip was not implanted. The contralateral kidney remained untouched. After 4 weeks of the surgical procedures, animals received water (SHAM and 2K1C) or aliskiren (10 mg/kg) (SHAM + A and 2K1C + aliskiren) daily via orogastric gavage for four weeks. The systolic blood pressure (SBP) was measured using the noninvasive method of plethysmography of the caudal artery (Letica LE 5001, Panlab, Barcelona, Spain) once a week during the all experimental periods.

On the day of euthanasia, eight weeks after the experimental period, the rats were anesthetized with intraperitoneal injection of ketamine (100 mg/kg) and xylazine (10 mg/kg), and the blood was removed by cardiac puncture and processed to obtain the plasma. Then, the adrenal glands and left kidneys were collected and fixed in 4% paraformaldehyde for light microscopy or 2.5% glutaraldehyde for electron microscopy analysis.

### 2.2. Immunohistochemical Analysis

The adrenal glands and left kidneys fixed with 4% paraformaldehyde were embedded in paraffin and sectioned (5 *μ*m thickness). Sections had antigen retrieval performed by citrate buffer at pH 6.0 and incubated for 30 min at 60°C. Endogenous peroxidase activity was blocked using 0.3% hydrogen peroxide (H_2_O_2_), and nonspecific binding of the polyclonal antibodies was blocked by incubation 5% (w/v) BSA. Subsequently, sections were incubated with antibodies, and these reactions were amplified using a biotin–streptavidin system (Dako, USA). Immunoreactive products were visualized using diaminobenzidine (DAB) reagent (Dako, USA) and counter-stained with hematoxylin. We used anti-AT1R (sc-515884), antirenin (sc-137252), anti-PGC1-*α* (sc-518025), anticytochrome C (sc-13156), anti-Bax (sc-20067), anti-Bcl-2 (sc-7382), anti-EEA1 (sc-137130), and Rab 7 (sc-81922) (dilution 1 : 200, Santa Cruz Biotechnology, CA, USA, for all) antibodies. The immunostaining of the zona glomerulosa adrenal was observed under a light microscope equipped with a CCD camera (Olympus BX53 with the camera Olympus DP72, Japan).

### 2.3. Morphometry

The expression of all markings were quantified in 20 randomly acquired fields, in the Image-Pro Plus 7.0 software, at a magnification of ×1000. The regions stained in the different analysis (AT1, EEA-1, Rab 7, Bax, BCL-2, cytochrome C, and PGC1-*α*) were measured, and the percentage of positive area was calculated (positive area/total area of the field).

### 2.4. Total Plasmatic Proteins Quantification

Three milliliters of blood were collected using a heparin revested syringe. The blood was centrifuged, and the plasma was collected. The total protein of the plasma was accessed by bicinchoninic acid assay (BCA) following the data sheet steps (ThermoFischer Scientific; #23227), and the measure was made in spectrophotometer at 562 nm.

### 2.5. Western Blotting for Plasmatic Renin Evaluation

The plasma renin was executed by Western blotting assay using 25 *μ*g of total protein. The sample was diluted in denaturized buffer (10% SDS, 0.1 M EDTA, 0.5 M Tris, 20% glycerol, and 0.01% bromophenol blue) and denatured at 95°C for 5 min. A final volume of 20 *μ*L was added to each well and submitted to electrophoresis in 12% of polyacrylamide gel with 80V for 90 min. The protein was then transferred to a nitrocellulose membrane in a semidry system for 50 min with 15V for each membrane. The protein transference was confirmed by membrane Pouceau staining, followed by block solution (2.5% milk; 1.37 M NaCl, 0.027 M KCl, 0.25 M Tris Base, 0.01% Tween 20, and pH7.4) for 1 h at room temperature and renin primary antibody overnight incubation (Santa Cruz Biotechnology; #SC-137252; 1 : 200). On the following day, the membrane was washed in T-BST buffer (1.37 M NaCl, 0.027 M KCl, 0.25 M Tris Base, 0,01% Tween 20, and pH 7.4) and incubated with anti-mouse antibody conjugated to HRP (Proteintech Group, Inc. Rosemont, USA; #SA00001-1; 1 : 2000) for 1 h at room temperature. The peroxidase was developed with PierceTM ECL Western Blotting Substrate (ThermoFischer Scientific, #32109) as described by the datasheet manufacturer. The renin expression and relative intensity of the band was analyzed through the ChemiDoc XRS + System (Bio-Rad Laboratories Inc.) using Image LabTM Software (Bio-Rad Laboratories Inc., Version 6.0.1 build 34, Standard Edition, 2017). The results were expressed as arbitrary unit/plasma total protein (*μ*g/*μ*L).

### 2.6. Ultrastructural Analysis

Adrenals were collected, cut into small tissue blocks (1 mm³), and immediately fixed in 2.5% glutaraldehyde and 0.1 M cacodylate buffer (pH 7.2) at 4°C. The fragments were postfixed in 2% osmium tetroxide and then dehydrated in an increasing series of acetone (30, 50, 70, and 100%) and embedded in Epon resin and polymerized at 60°C for 72 hours. Ultrathin sections (70 nm) were contrasted with uranyl acetate and lead citrate and then examined using a JEOL 1001 transmission electron microscope (JEOL Ltd., Tokyo, Japan) at 80 kV. For the quantification of mitochondria, six images were randomly captured in cells of the zona glomerulosa of the all experimental groups, at a magnification × 25.000 of the transmission electron microscope [28] (Miranda et al.).

### 2.7. Statistical Analysis

Data were analyzed with GraphPad Prism software (v 6.0, San Diego, California, USA) using two-way ANOVA with the Holm–Sidak posttest. All results are presented as the mean ± standard deviation, and the differences between groups were considered statistically significant when values of *p* < 0.05.

## 3. Results

### 3.1. Assessment of Systolic Blood Pressure and AT1R and Renin Expression

**Figure 1:**
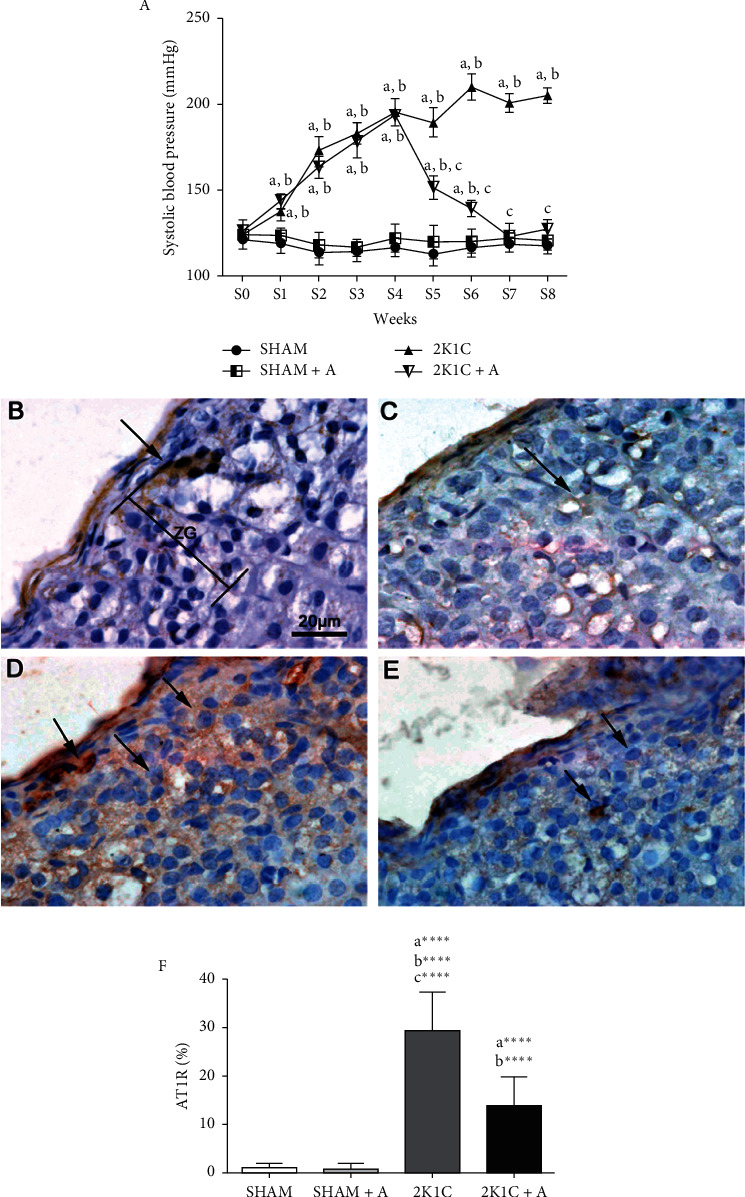
Evolution of blood pressure and detection and quantification of AT1R expression. (A) Evolution of SBP in millimeters of mercury (mmHg). Detection of AT1R expression by immunohistochemistry in the (B) SHAM group, (C) SHAM + A, (D) 2K1C, and (E) 2K1C + A. (F) Quantification of AT1R expression in the zona glomerulosa of the adrenal gland of all experimental groups. Arrows indicate the immunostained positive cells. ZG, zona glomerulosa. (a) represents ≠ to the SHAM group, (b) represents ≠ to the SHAM + A group, and (c) represents ≠ to the 2K1C + A group. ^*∗∗∗∗*^*p* < 0.0001. *n* = 10 for all experimental groups.

The systolic blood pressure (SBP) of all groups was accompanied throughout the treatment. SBP of the SHAM and SHAM + A groups remained constant throughout the experiment (SHAM: 117 ± 5 mmHg and SHAM + A: 120.6 ± 5 mmHg) and did not show significant differences between them until the end of the experiment. On the other hand, the SBP of the animals of the 2K1C group increased gradually from week 1 to week 8 (from 136.8 ± 5 mmHg to 205.5 ± 4 mmHg). In the first four weeks, animals in the 2K1C + A group showed a gradual increase in SBP levels (from 142.6 ± 3 mmHg to 193.2 ± 2 mmHg). At week 4, when the aliskiren administration started, the animals in the 2K1C + A group showed a reduction in SBP (from 193.2 ± 2 mmHg to 150.4 ± 5 mmHg), and in week 7, the SBP levels of these animals were similar to the SBP levels of the SHAM and SHAM + A animals (123.2 ± 3 mmHg) (Figure 1(A)). In an immunohistochemical study, positive anti-AT1R antibody immunostaining was observed in all experimental groups in the zona glomerulosa of the adrenal gland (Figures 1(B)–1(E)). The SHAM group (Figure 1(B)) presented an area of 0.91 ± 0.17% stained and the SHAM + A group 0.66 ± 0.19% (Figure 1(C)). The 2K1C group (Figure 1(D)) showed a significant increase of AT1R expression (30.01 ± 2.71%), which was reduced in the 2K1C group treated with aliskiren (Figure 1(E)) (13.67 ± 1.86%). The quantification of AT1R is shown in Figure 1(F).

**Figure 2:**
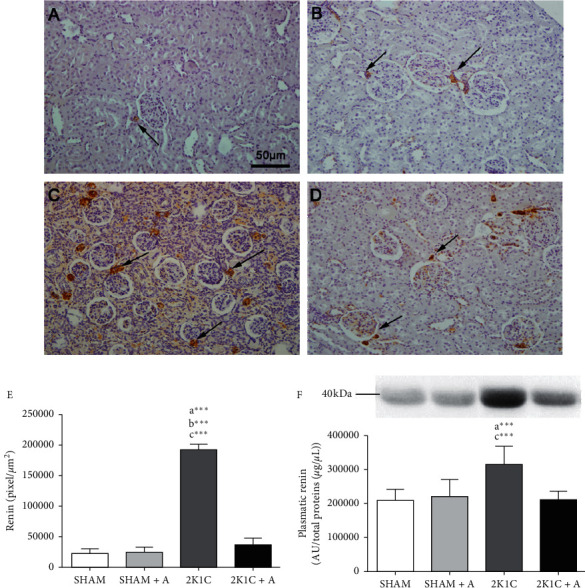
Detection and quantification of renin in renal tissue and plasma. Detection of renin expression by immunohistochemistry in the Sham group (A), Sham + A (B), 2K1C (C), and 2K1C + A (D). Arrows indicate positive immunostaining in juxtaglomerular cells. Quantification of renin expression in juxtaglomerular cells of hypertensive and control groups (E). Evaluation of the plasma renin expression in all groups (F). (a) represents ≠ to the SHAM group, (b) represents ≠ to the SHAM + A group, and (c) represents ≠ to the 2K1C + A group. ^*∗∗∗∗*^*p* < 0.0001. *n* = 10 for all experimental groups.

In order to confirm that the hypertension process led to an increase in active renin expression, we performed an immunohistochemical study, in which positive antirenin antibody immunostaining was observed in the juxtaglomerular cells of the renal cortex in all experimental groups (Figures 2(A)–2(D)). The 2K1C group (Figure 2(C)) showed more positive areas, both in the juxtaglomerular cells and in the tubular region, compared to the Sham (Figure 2(A)) and Sham + A (Figure 2(B)) groups. On the other hand, a decrease in the positive areas was observed in the 2K1C group treated with aliskiren (Figure 2(D)) compared to the 2K1C group. By quantifying the antirenin antibody, a significant increase in the number of cells marked in the 2K1C group was observed in relation to the other groups, as showed in Figure 2(E). The analysis of renin expression in plasma showed that this protein increased in rats of the 2K1C group (314805.64 ± 52676.3) compared to both Sham (209505.86 ± 32128.61) and Sham treated with aliskiren (220639.17 ± 44924.08). The treatment of 2K1C rats with aliskiren (210937.59 ± 25367.86) reduced the renin expression compared to nontreated animals. Renin was detected with the size referring to its active form, that is, with 40 kDa (Figure 2(F)).

### 3.2. Evaluation of the Expression of Early and Late Endosomes, Related to Autophagy in the Zona Glomerulosa

**Figure 3:**
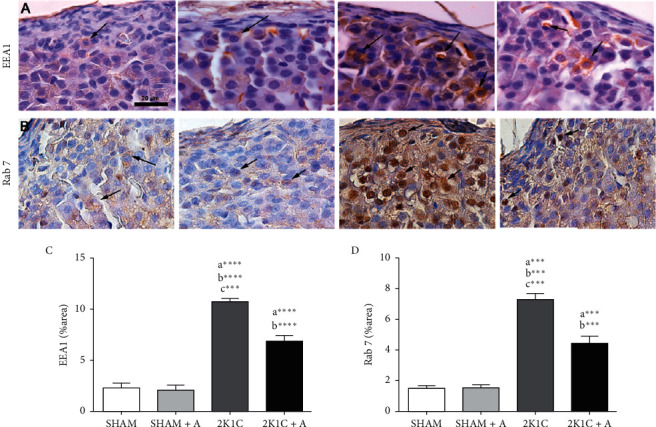
Detection and quantification of EEA1 and Rab 7 expression by immunostaining in the zona glomerulosa. (A)-(B), photomicrographs of all experimental groups marked with anti-EEA1 and anti-Rab 7 antibodies, respectively (C)-(D), quantification of EEA1 and Rab 7, respectively. Arrows indicate the immunostained positive cells. (a) represents ≠ to the SHAM group, (b) represents ≠ to the SHAM + A group, and (c) represents ≠ to the 2K1C + A group. ^*∗∗∗∗*^*p* < 0.0001; ^*∗∗∗*^*p* < 0.001. *n* = 10 for all experimental groups.

The detection of early endosomal antigen 1 (EEA1) and Rab 7 (late endosome marker) was also performed by immunohistochemistry (Figures 3(A)–3(B)). In the both evaluation of EEA1 and Rab 7, the 2K1C group showed a significant increase of 5-fold in the expression of these endosomes compared to both control groups. In the 2K1C + A group, a significant decrease of expression was observed compared to the 2K1C group (10.74 ± 0.32–6.88 ± 0.51% in EEA1 and 7.35 ± 0.34–4.41 ± 0.46% in Rab 7) (Figures 3(C) and 3(D)).

### 3.3. Regulator Proteins Evaluation of the Apoptotic Process in the Zona Glomerulosa by Immunohistochemistry

**Figure 4:**
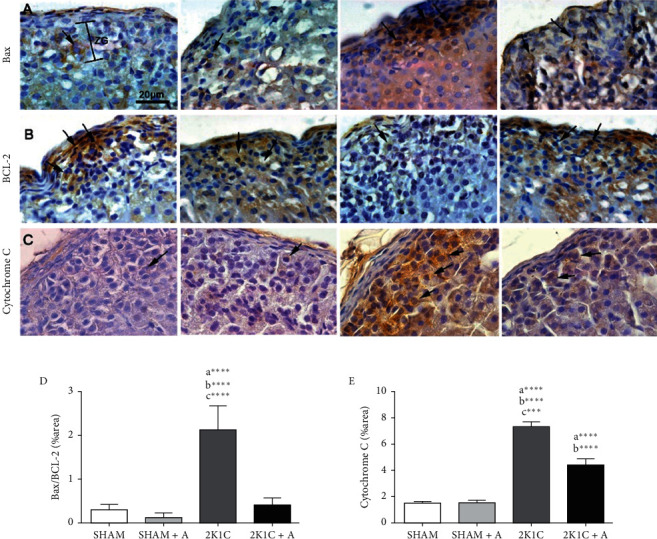
Detection and quantification of Bax, BLC-2, and cytochrome C expression in the zona glomerulosa. (A–C) Photomicrographs of all experimental groups are presented stained with anti-Bax, anti-BCL-2 and anticytochrome antibodies, respectively. (D-E), quantification of Bax and BLC-2 ratio and cytochrome C proteins are shown, respectively. ZG, zona glomerulosa. Arrows indicate the immunostained positive cells. (a) represents ≠ to the SHAM group, (b) represents ≠ to the SHAM + A group, and (c) represents ≠ to the 2K1C + A group. ^*∗∗∗∗*^*p* < 0.0001;^*∗∗∗*^*p* < 0.001. *n* = 10 for all experimental groups.

The detection of Bax, BCL-2, and cytochrome C were observed in a immunostaining performed for all groups (Figures 4(A)–4(B)). In the analysis of the expression of proapoptotic and antiapoptotic proteins (Bax and BCL-2, respectively), as expected, there was no significant difference between animals in the SHAM and SHAM + A groups in expression the Bax and BCL-2 proteins (Figure 4(D)). The 2K1C group showed a significant increase in Bax protein expression and decreased BLC-2 protein compared to the other experimental groups (Figure 4(D)). On the other hand, the 2K1C group treated with aliskiren showed a decrease and an increase in the expression of Bax and BLC-2 proteins, respectively, compared to the 2K1C group (Figure 4(D)). The ratio of proapoptotic and antiapoptotic proteins is shown in Figure 4(D) and summarize the results described above, indicating a proapoptotic process in the cells of the zona glomerulosa of the adrenal cortex of animals in the 2K1C group, which was reversed by treatment with aliskiren. In mammals, the release of cytochrome C from mitochondria after the Bax increase stimuli is considered a primary activator of the apoptosis across of caspase cascade, so we also evaluate their expression in the different groups. There was no significant difference between animals in the control groups. The 2K1C group showed a 5-fold increase of the cytochrome C expression compared to the SHAM and SHAM + A groups (Figure 4(E)). On the other hand, the 2K1C + A group presented a significant decrease compared to the 2K1C group (8.05 ± 0.32–4.89 ± 0.49%) (Figure 4(E)).

### 3.4. Evaluation of the Zona Glomerulosa Ultrastructure and Mitochondrial Biogenesis by PGC1-*α* Expression

**Figure 5:**
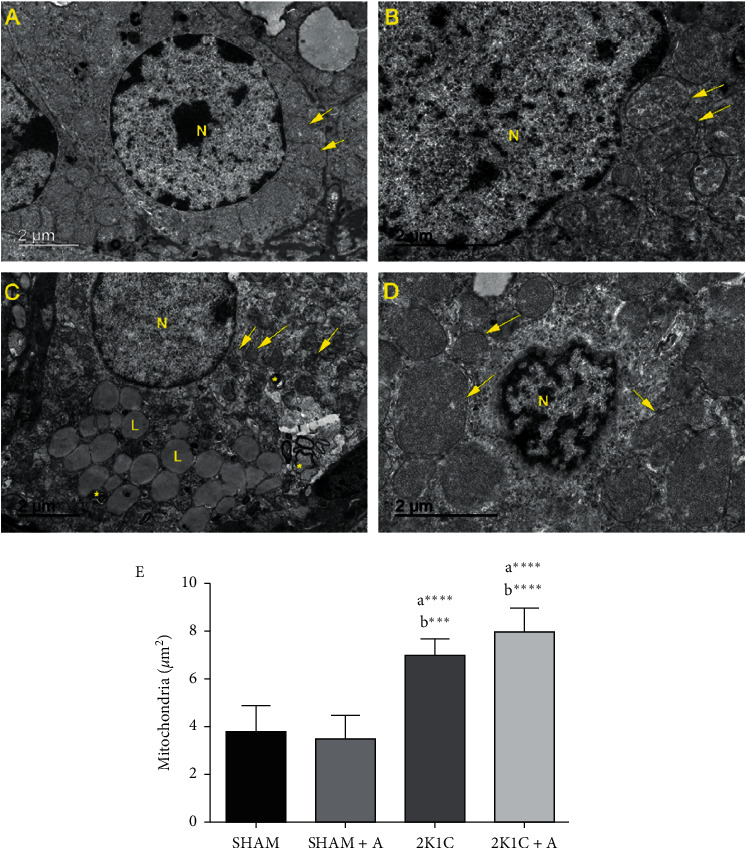
Analysis of zona glomerulosa ultrastructure. Experimental groups: (A) SHAM; (B) SHAM + A; (C) 2K1C; (D) 2K1C + A. The nuclei (N), mitochondria (arrows), lamellar endosomes/lysosomes (^*∗*^), and the lipid bodies (L) are indicated. Quantification of the number of mitochondria in zona glomerulosa of all experimental groups (E). (a) represents ≠ to the SHAM group and (b) represents ≠ to the SHAM + A group. ^*∗∗∗∗*^*p* < 0.0001; ^*∗∗∗*^*p* < 0.001. *n* = 10 for all experimental groups.

In the analysis of the adrenal ultrastructure (Figure 5), the SHAM and SHAM + A groups (Figures 5(A) and 5(B), respectively) presented preserved nuclei (*N*) and mitochondria (arrows) displaying normal features with tubular cristae. In the 2K1C group (Figure 5(C)) was observed an electrondense nucleus and many differences in mitochondria, especially. These organelles were swollen, with electrondense deposits in the matrices, with lamellar and/or circular concentric cristae, and ruptured mitochondrial membrane. In addition, we observed the presence of lipid bodies and lamellar endosomes/lysosomes, which were formed by sequestering a portion of the cytoplasm for digestion, corroborating the EEA1 and Rab 7 findings in the immunostaining analysis. In the 2K1C + A group (Figure 5(D)), we observed nuclei with preserved structure, the presence of well-developed mitochondria with intact membranes devoid of lamellar or circular crystals, indicating that the treatment with aliskiren was able to reverse the ultrastructural changes evidenced in the 2K1C group. Quantification of the number of mitochondria was performed using electromicrography of all experimental groups (Figure 5(E)). The 2K1C and 2K1C + A groups presented a significant increase in the number of mitochondria in relation to the other groups.

**Figure 6:**
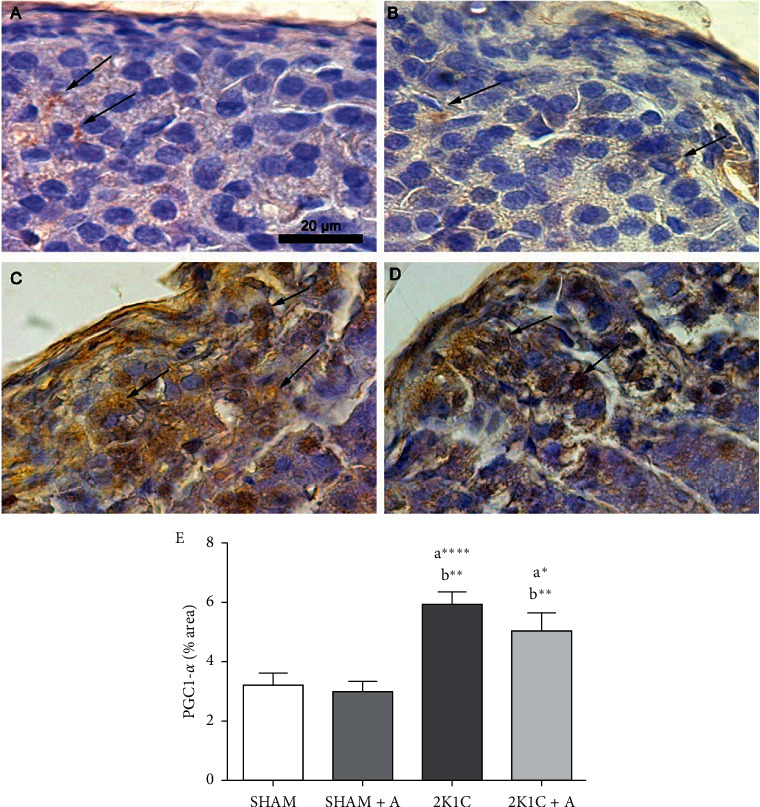
Detection of PGC1-*α* by immunostaining and quantification in the zona glomerulosa. Photomicrographs of groups (A) SHAM; (B) SHAM + A; (C) 2K1C; and (D) 2K1C + A. (E) Quantification of PGC1-*α* expression in the zona glomerulosa of all experimental groups. Arrows indicate the immunostained positive cells. (a) represents ≠ to the SHAM group, (b) represents ≠ to the SHAM + A group, and (c) represents ≠ to the 2K1C + A group. ^*∗∗∗∗*^*p* < 0.0001, ^*∗∗*^*p* < 0.01, and ^*∗*^*p* < 0.05. *n* = 10 for all experimental groups.

Based on the results found in the ultrastructural analysis, we performed an immunostaining for the PGC1-*α* protein, related to mitochondrial biogenesis. Both SHAM and SHAM + A groups (Figures 6(A) and 6(B), respectively) showed little staining in the specific labeling for PGC1-*α*. The 2K1C and 2K1C + A groups showed an increase of approximately twice in the PGC1-*α* expression compared to the SHAM and SHAM + A groups (Figures 6(C)–6(E)). The 2K1C + A group showed no difference compared to the 2K1C group (Figures 6(C)–6(E)).

## 4. Discussion

To the best of our knowledge, this study is the first to compile the possible alterations in apoptosis, autophagy, and mitochondrial biogenesis processes in the adrenal gland in rats with the 2K1C model. This experimental renovascular hypertension model is used widely to delineate the relationship among the renin-angiotensin system and hypertension and its local and systemic changes and test the effect of therapeutic drugs in the treatment of chronic hypertension [12–16].

Aliskiren, the first effective direct oral renin inhibitor approved for the treatment of hypertension, acts by suppressing the first and rate-limiting step of the RAAS leading to more complete blockade of this system. Previous studies have demonstrated the potential for aliskiren in reducing plasma renin and angiotensin II activity and aldosterone levels, leading to lower blood pressure [4, 29]. Additionally, this antihypertensive reduces remodeling, inflammation, stress-oxidation, and fibrosis in different tissues [27, 30].

Here, surgical procedures developed by Goldblatt in 1934 [12] for placing the silver clip around the left renal artery of rats showed positive results, once 2K1C rats developed sustained hypertension starting 2 weeks after the clipping, characterizing renovascular hypertension. The administration of aliskiren was effective with a significant reduction in SBP, as observed in other studies [12, 31, 32].

Additionally, the treatment reduces the AT1R expression in the adrenal cells of the zona glomerulosa in hypertensive rats. In this study, the 2K1C rats showed, as expected, significantly increased AT1R expression compared to the SHAM and SHAM treated with aliskiren groups, which is a physiological condition caused by the RAAS deregulation [33]. Indeed, increased AT1R expression was already observed in the zona glomerulosa of the adrenal gland and kidney in a study performed by Mansour and collaborators [34], in rats with diabetic nephropathy, caused by RAAS deregulation and over activation. The AT1R is an integral membrane protein that mediates most of the pathological and physiological effects associated with angiotensin II. When angiotensin II binds to these receptors, widely distributed throughout the body, it results in vasoconstriction, inflammation, oxidative stress, apoptosis, growth factor responsible for cell proliferation, and cardiovascular and renal effects, and in the adrenal cortex, this pathway leads to aldosterone release by cells in the zona glomerulosa [33, 35, 36]. We show herein that, in the treated 2K1C group, an increase observed on AT1R expression in the hypertension group was reversed effectively; therefore, we investigated the effect of aliskiren in some AT1R-mediated pathways.

The evolution of renovascular hypertension has been described as having the following three stages or phases. It is well accepted that the initial phase of the renovascular hypertension is mediated by the renin-angiotensin system (RAS) and occurs in approximately 4 weeks after clipping the kidney. During this phase, there is a rise in renin secretory rate, plasma renin activity (PRA), and systemic blood pressure [37]. In our study, the development of renovascular hypertension occurred in 4-5 weeks after clipping the kidney, being in a period between the initial and intermediate phase (or salt-retention phase). We detected active renin (with 40 kDa) in juxtaglomerular cells, quantified its expression in the kidney and plasma, with a greater detection in the hypertensive group, which was greatly reduced with the treatment. Although, the measurement of aldosterone concentration was not performed, being a limitation of our study, many studies in the literature with the same or higher period of development of renovascular hypertension, showed elevations in plasma renin activity (PRA) and circulating angiotensin II (ANG II) that lead to increased aldosterone [38–41]. Additionally, Nurfaradilla et al. [42] described that in the male Sprague-Dawley 2K1C hypertension model (4 weeks), the plasma renin level, serum angiotensin-converting enzyme (ACE) activity, and plasma angiotensin II level were significantly elevated in the 2R1C group compared to the SHAM group indicating that the increased plasma renin level was likely by increasing renal production in response to loss of perfusion. Gromotowicz-Poplawska et al. [43] showed that PRA and aldosterone was significantly higher in 2K1C rats compared to Sham rats after 6 weeks of clipping of the kidney. Bearing in mind the above data, excessive activation of the renin-angiotensin-aldosterone system leads to a loss of vessels dilatability and consequent chronic reduction of renal blood flow, leading to increased renin release and, finally, aldosteronism [44, 45]. What is more, the salt and water retention by the clipped kidney and contralateral GFR falls to or below normal may trigger the overproduction of Ang II leading to production of aldosterone by the zona glomerulosa of the adrenal glands. In fact, data from our laboratory showed that these animals in the 2K1C group showed reduced urinary volume and decreased creatinine and urinary urea, showing impaired renal filtration capacity, renal damage, and water retention in these animals [27]. Ang II can affect the expression of hormonal regulators of stress, such as corticosterone [46]. However, plasma corticosterone evaluated in rats induced to hypertension by the 2K1C model for 4 weeks showed no significant changes in the 2K1C group [47]. In another study, Müller et al. [48] showed that there was no change in the basal level of corticosterone after systemic treatment of rats with exogenous angiotensin. Weissheimer et al. [47] suggest the activation of compensatory mechanisms in response to the higher plasma Ang II preventing increases in plasma corticosterone in 2K1C animals. Finally, several studies have already shown that aliskiren was effective in reducing PRA [49], Ang I, and plasma Ang II concentrations during renovascular hypertension [50].

In 2K1C rats, the AT1R-NADPH oxidase pathway induces renal oxidative stress [19] contributing to the development of oxidative stress in various organs [51]. Overproduction of reactive oxygen species (ROS) is involved in autophagy activation [52]. Therefore, we investigated the expression of EEA1 (early endosomal antigen 1) and Rab 7, (a later endosome marker protein) markers of membrane involved in vesicle-mediated transport between organelles, including the formation and control of autophagosome [53, 54]. EEA-1 is a Rab 5 effector. The replacement of Rab5 by Rab 7 characterizes the transformation of early endosome to endosome late and so the activation of the autophagy process [55]. Our results showed a significant increase in EEA1 and Rab 7 expression in the animals of the 2K1C group compared to the treated SHAM and SHAM groups, indicating that the autophagy process is active in cells of the zona glomerulosa during the hypertensive process. In steroid-secreting cells, as in the adrenal gland, autophagy target is the steroid-producing organelles being able to take, to dysfunction and degradation [56]. The aliskiren-treated hypertensive group showed a reduction in both markers, indicating that this drug has an inhibiting effect on autophagy, which may be related the AT1R-NADPH oxidase pathway, since there was a reduction in AT1R expression in this group.

The autophagic process has a great relationship with apoptosis; therefore, we analyzed whether the zona glomerulosa of hypertensive animals could be in the process of cell death [57]. The expression of proteins related to the apoptotic process showed that, in the 2K1C group, there was a significant increase in the expression of cytochrome C and Bax compared to the other groups, suggesting apoptosis. In mitochondrial dysfunction caused in Bax increase, the permeabilization of the mitochondrial membrane can disrupt the electron transport chain and affect the cytochrome C function. In response, cytochrome C is released to the cytosol that leads to apoptosis. This release can be mediated by proapoptotic Bax and antiapoptotic Bcl-2 proteins [58, 59]. In studies carried out in cardiac myocytes of patients with heart failure, the expression of the protein from the Bax protein was increased, while the Bcl-2 expression was decreased, resulting in a decrease in the Bax/Bcl-2 ratio, indicating that cell apoptosis was increased in cardiac myocytes [60]. A study conducted by Khalil [61] showed that in the adrenal cortex of rats treated with the ketaconazole drug and a significant decrease in aldosterone and plasma corticosterone, the zona glomerulosa was reduced and the fasciculate zone increased. This study also observed a significant increase in the immunostaining of Bax expression in the cells of the zona glomerulosa, suggesting a marked change in the morphology of the adrenal cortex. In our study, the aliskiren treatment was able to reduce this protein expression, such as cytochrome C, which means a decrease in the damage and cell death caused by hypertension in the zona glomerulosa.

Besides, the ultrastructural analysis of adrenal cells in the zona glomerulosa showed that the animals of the 2K1C structure presented important changes, such as endosomes, dense electron nuclei, and mitochondrial alterations. These ultrastructural characteristics may suggest the beginning of the apoptotic process, possibly due to the activation of the autophagy, as observed in this study. We also observed that only the hypertensive group had a large accumulation of lipid corpuscles. This accumulation of lipids probably occurs due to an overexpression of aldosterone in hypertensive animals, which was reversed by treatment.

Similarly, a study showed an increase in lipids in hypertensive animals, which was decreased in the groups treated for hypertension with Zn and Cu [62]. Additionally, we found in the hypertensive group several double-membrane lamellar lysosomes, which are responsible for digestion of cytoplasm and organelles. This is one of the responses to aldosterone excess that activates the autophagy system in order to remedy the oxidative stress caused by hypertension [63]. In our study, these lysosomes were not observed after treatment with aliskiren, showing that the drug is able to reverse the stress caused by hypertension, leading even to the reduction of autophagic bodies. The mitochondria of the 2K1C group were well developed, with dense electron deposits in the mitochondrial matrices, displaying concentric lamellar and/or circular crests and ruptured membrane, suggesting severe mitochondrial dysfunction. Some of these changes were also observed in 2K1C treated with aliskiren rats, but the nuclei were preserved, no endosomes were observed, and we noticed the presence of mitochondria with intact membrane. These similar changes have been reported in cells of the glomerular and fasciculate zones of rats after induction of adrenal stress caused by liver ischemia [3]. Florea and Crãnciun [64] also reported the same ultrastructural changes in the cells of the zona glomerulosa of rats after treatment with toxins that inhibited mitochondrial activity. Mitochondria are organelles responsible for several metabolic functions, including energy production via oxidative phosphorylation. They are also a major source of reactive oxygen species (ROS), and their overproduction damages the mitochondrial DNA and the oxidation respiratory chain, causing mitochondrial dysfunction [65, 66].

The PGC1-*α* protein potently modulates mitochondrial biogenesis and function and is also an important regulator of diverse metabolic pathways in response to environmental and physiological changes. It is not surprising that disorders of its function or expression is related to several diseases [67, 68]. In our study, we observed an increase in the expression of this protein in hypertensive animals, which was slightly lower in the treated hypertensive group, showing that there is a decrease in the need for mitochondrial biogenesis after treatment. Previous studies showed an overexpression of PGC1-*α* in mice with glomerulosclerosis compared to control animals, and this was due to a possible protective effect in order to restore mitochondria and the normal metabolic process [66]. Studies show that although the high expression of PGC1-*α* is beneficial for certain tissues, it can also be harmful in others [69–71]. A study in rats treated with aldosterone suggested that aldosterone may be contributing to mitochondrial dysfunction and oxidative stress in cardiac tissue, as the expression of PGC1-*α* and mitochondrial DNA was reduced compared to the control group, favoring the increase oxidative stress and the development of mitochondrial dysfunction [71]. In this study, in a contradictory way, the 2K1C rats showed a significant increase in the expression of PGC1-*α*, suggesting that the cells of the glomerulosa zone are undergoing metabolic stress and suggests that there is an attempt to recover the mitochondria to maintain local homeostasis.

## 5. Conclusion

In conclusion, this work demonstrated that the treatment with aliskiren is not only capable of reducing blood pressure but also able to improve the damage caused by hypertension in the zona glomerulosa of the adrenal glands, decreasing the autophagy, apoptosis, and ultrastructural injury. This study is the first to directly associate these parameters in 2K1C hypertension as the trigger for glomerular adrenal stress, and further studies are needed to elucidate this association, since this organ is very important, but it is still neglected.

## Data Availability

The data used to support this study are available within this article.
